# A New Scale for Rating Oral Health-Related Quality of Life in Denture Wearers

**DOI:** 10.3290/j.ohpd.b4997051

**Published:** 2024-02-20

**Authors:** Milica Jovanović, Slobodan Janković, Anđela Milojević Šamanović, Refet Gojak, Branislava Raičević, Jelena Erić, Marko Milosavljević

**Affiliations:** a Postgraduate Student, Teaching Assistant, University of Kragujevac, Faculty of Medical Sciences, Department of Dentistry, Kragujevac, Serbia. Hypothesis, experimental design, gathered and analyzed the data in partial fulfillment of requirements for the PhD degree, wrote the manuscript.; b Professor, University of Kragujevac, Faculty of Medical Sciences, Department of Pharmacy, Kragujevac, Serbia. Idea, performed the statistical analysis, wrote the manuscript.; c Assistant Professor, University of Kragujevac, Faculty of Medical Sciences, Department of Dentistry, Kragujevac, Serbia. Gathered and analyzed the data, proofread the manuscript.; d Assistant Professor, University of Sarajevo, Faculty of Medicine, Sarajevo, Bosnia and Herzegovina. Consulted on and performed statistical evaluation, proofread the manuscript.; e Postgraduate Student, University of Kragujevac, Faculty of Medical Sciences, Department of Pharmacy, Kragujevac, Serbia. Proofread the manuscript, contributed substantially to discussion.; f Lecturer, College of Dental Medicine, Qatar University, Qatar. Proofread the manuscript, and contributed substantially to discussion.; g Assistant Professor, University of Kragujevac, Faculty of Medical Sciences, Department of Dentistry, Kragujevac, Serbia. Experimental design, proofread and wrote the manuscript.

**Keywords:** complete denture, oral health, partial denture, quality of life

## Abstract

**Purpose:**

When carrying out prosthetic rehabilitation of edentulous and partially edentulous patients, great attention is paid to the personal attitude of the patients, their satisfaction with oral health and psychosocial interaction due to tooth loss, as well as the treatment of the resulting disorders. This attention has led to the development of various instruments for examining the quality of life related to oral health. The aim of this study was to develop and validate a reliable instrument in the Serbian language suitable for measuring oral health-related quality of life in patients who have been rehabilitated with complete or partial dentures.

**Мaterials and Methods:**

The study was unicentric and cross-sectional, and assessed the reliability and validity of a newly developed instrument for measuring the oral health-related quality of life in denture wearers (OHRQoL-DW). It was conducted on a sample of 200 adults from Serbia, wearers of various types of dentures, with a mean age 66.9 ± 10.3 years and male/female ratio of 86/114 (43%/57%).

**Results:**

The definitive version of the OHRQoL-DW scale with 28 items showed very good reliability, with Cronbach’s alpha = 0.938. Good temporal stability of the questionnaire was demonstrated, and satisfactory results were obtained for divergent and convergent validity tests. Exploratory factorial analysis revealed four domains of oral health-related quality of life in denture wearers: physical, psychosocial, environmental and aesthetic.

**Conclusions:**

The OHRQoL-DW scale is a reliable and valid generic instrument for measuring the oral health-related quality of life in patients wearing dentures, which is one of the most important outcomes of oral health in prosthetic treatment.

Oral health-related quality of life (OHRQoL) is part of the general health status, and it is also used as an instrument in dental epidemiological and clinical studies that measure the extent of tooth loss and the impact of dental prosthetic interventions.^23^ Tooth loss significantly affects the performance of the basic functions of the stomatognathic system, such as chewing, swallowing and speaking. In addition to difficulty in performing those functions, in patients who do not have one or more of their teeth, aesthetics, facial expressions and quality of life in general can be significantly impaired. Different reactions can also occur that are related to the psychological state of the person and refer to emotional distress, depression and anxiety.^18^ On the other hand, suboptimal oral health can greatly affect social interactions, causing avoidance of social contacts, impairing success in finding a partner and even employment.^11^

Any kind of prosthetic treatment of edentulous and partially edentulous patients helps to overcome aesthetic problems, but above all to rehabilitate impaired or lost oral functions, which is confirmed by numerous studies.^2,7,17^ Parallel to the goal of achieving adequate prosthetic rehabilitation of the patients, great attention is paid to their personal attitude, their satisfaction in life and psychosocial interactions due to tooth loss, as well as the treatment of the resulting disorders. This focus has led to the development of various instruments for examining quality of life related to oral health. OHRQoL is defined as a multidimensional construct comprising physical, social and emotional aspects of oral health and its impact on everyday life.^25^

The most commonly applicable instruments for examining the quality of life related to oral health are questionnaires, among which the Oral Health Impact Profile-49 (OHIP-49) stands out. This questionnaire contains 49 items that are based on the theoretical model developed by the World Health Organization (WHO), while Locker’s model of oral health was used to define the impact of seven conceptual dimensions: functional limitations (e.g., difficulty chewing), physical pain (e.g., tooth sensitivity), psychological discomfort (e.g. self-consciousness), physical limitations (e.g., changes in diet), psychological limitations (e.g., reduced concentration), social limitations (e.g., avoidance of social interactions) and handicap (e.g., inability to work productively).^14,25^ According to the Likert scale, each question in the questionnaire measures the severity of oral distress felt by the patient in the past twelve months (the score ranges from 0 to 196; a higher score indicates worse quality of life related to oral health).^20^

A shortened version of the OHIP-49 questionnaire is the OHIP-14, which was developed later with the aim of reducing the time required to fill out the questionnaire as well as the number of incomplete answers.^24^ Another version of the OHIP-49 that was later developed and applied worldwide is the OHIP-EDENT questionnaire, which contains 19 questions and is adapted to edentulous patients and patients rehabilitated with complete dentures.^1^ These questionnaires have been translated into several languages and validated in different populations, including Serbian. However, the above-mentioned questionnaires were not developed specifically for rating oral health in a population of edentulous and partially edentulous adults who wear different types of dentures.

The aim of this study was to develop and validate a reliable scale suitable for measuring OHRQoL in edentulous and partially edentulous patients who have been treated with complete or partial dentures.

## Materials and Methods

### Study Design

The study was designed as unicentric, cross-sectional research, and was approved by the Ethics Committee of the Faculty of Medical Sciences, Kragujevac, Serbia (No 01-3101, date 23.03.2022). Before the study began, the participants completed an informed consent form, and they received the respect and care they deserved in accordance with the Declaration of Helsinki’s tenets.

### Construction and Validation of the New Scale

The new scale (Oral Health-Related Quality of Life in Denture Wearers – OHRQoL-DW) was developed following the guidelines set by DeVellis^6^ in eight steps.

(1) The health-related quality of life (HRQoL) in adults wearing any kind of dentures was determined as the object of measurement, and it was theoretically based on a study by Bekiroglu et al^3^ which investigated oral complaints of denture-wearing elderly people living in nursing homes.

(2) The item pool with completely new items was generated during two brainstorming sessions, one week apart; only authors participated in the sessions and creation of the item pool, taking care to cover oral complaints of denture-wearing elderly people identified by Bekiroglu et al. Members of the group that generated the items had the following profiles: three prosthetic dentistry specialists and one clinical pharmacology specialist.

(3) The Likert scale was chosen as the format of measurement, with the following possible answers to statements reflecting OHRQoL: “I disagree completely”, “I disagree partially”, “I neither agree nor disagree”, “I agree partially”, and “I agree completely”. The answers were rated from 5 “I disagree completely” to 1 “I agree completely”.

(4) The initial pool of items was revised and corrected by the three-member expert committee composed of a prosthetics specialist, an endodontic specialist and a clinical pharmacology specialist. These profiles were chosen to broadly cover both prosthetic and non-prosthetic aspects of oral health, since the scale was intended for use primarily at the secondary and tertiary healthcare level.

(5) In order to discover socially desirable behaviour of respondents, one validation item was included in the questionnaire: “I always treat the others with respect.”

(6) The initial pool of items was tested on 20 to 30 patients for clarity and comprehension, and after the pilot phase and a few minor changes, final versions of the questionnaire were copied into the Serbian language, and prepared for reliability testing.

(7) The items were evaluated.

(8) The questionnaire length was optimised.

The cognitive status of the study subjects was assessed by independent specialists of prosthetic dentistry who treated study subjects within the framework of regular prosthetic care. For the purpose of convergent validation of the OHRQoL-DW the study subjects were offered the Questionnaire Evaluating the Impact of Prosthetic Dental Treatments on Patients’ Oral Health Quality of Life (PDT-OHQoL) in Serbian.^16^ Divergent criteria validation was made by the 10-item Emotional Regulation Questionnaire (ERQ) in Serbian, which measures two strategies of emotional regulation: cognitive reappraisal and emotion suppression.^19^ Permission to use these supplementary questionnaires was obtained from the authors before the start of the study.

### Data Collection

Final Serbian versions of both the new (OHRQoL-DW) and accompanying (PDT-OHQoL and ERQ) questionnaires were tested for reliability on a convenience sample of adult outpatients of the Dentistry Institute, Faculty of Medical Sciences, Kragujevac, Serbia, who had been wearing dentures of any kind for more than 1 month. The surveys took place from 1 April 2022 to 15 May 2023. The inclusion criteria were wearing any type of denture ≥ 1 month, no cognitive impairment, literacy, and age over 18. Pregnancy, nursing, serious psychiatric illnesses (such as mood disorders, psychoses, and mental retardation), usage of psychotropic medicines, long-term alcohol abuse, and emergency situations were among the exclusion criteria. All participants who came into contact with an investigator on the survey day (and who met the inclusion criteria/ did not meet the exclusion criteria) were given the questionnaires. This sample of participants was sequential. One copy of the questionnaire was filled out during the initial interview by the researchers who were interviewing the study participants, and a second copy was provided to the participants to fill out the following day on their own at home and return it to the researchers. The second encounter was 15 to 30 days later, at which time both OHRQoL-DW and accompanying scales were completed by the researchers interviewing the patients to assess temporal stability.

### Data Analysis

The reliability of the OHRQoL-DW scale was tested threefold. First, the entire questionnaire’s internal consistency was evaluated by computing Cronbach’s alpha. Second, Cronbach’s alpha was determined for each of the two sides of the questionnaire, each of which had the same number of items. The “prediction” formula was used to determine the Spearman-Brown coefficient for the entire questionnaire based on the alphas of both portions, the number of questions, and the average correlation between questions in the two parts.^27^ Third, mean scores and their variations were calculated for each question to see if it was possible to gauge the whole range of probable responses.

The questionnaire underwent an exploratory factor analysis to identify key factors.^9^ In principal axis factoring, a scale’s items are grouped together according to fewer factors that account for the majority of the variance in the responses to the scale’s items. The maximum variance-covering factors are maintained, while the rest are eliminated. The eigenvalue of each factor indicates how much variance it can explain. The Kaiser-Meyer-Olkin measure of sampling adequacy and Bartlett’s test of sphericity were used to test the sample’s results against the assumptions of the factor analysis. The factors were then initially extracted without rotation using a Scree-plot and the generalised least-squares extraction method, conditional on eigenvalues > 1.0. Second, using the Varimax approach, the referent axes were rotated, and another extraction was performed. The calculations were performed by SPSS statistical software, version 25.0 (IBM; Armonk, NY, USA).

Content validity of the questionnaire was evaluated by an independent panel of three experienced clinicians at the Dentistry Institute, Faculty of Medical Sciences, Kragujevac, Serbia: a prosthetics specialist, an endodontics specialist, and a clinical pharmacology specialist.

The criterion validity was tested by two methods: 1) comparing the OHRQoL-DW score with the PDT-OHQoL score (convergent validity testing), and 2) comparing the OHRQoL-DW score with the score of the Emotional Regulation Questionnaire (ERQ) (divergent validity testing). The correlations between scores of the questionnaire’s values were calculated. The calculations were performed using SPSS.

Comparing the OHRQoL-DW scores of study participants with various types of dentures allowed the external validity of the study to be evaluated. The Mann-Whitney U-test was used to compare the data. The researchers regularly interviewed the study participants 15 to 30 days following the initial meeting in order to test the temporal stability of the OHRQoL-DW results. After the first encounter with the study participants, a second one was planned.

## Results

The OHRQoL-DW scale was composed of 28 questions, and after the pilot phase, minor adjustments were tested on the sample of 200 study subjects. The mean age was 66.9 ± 10.3 years; the mean body weight was 78.02 ± 13.58 kg; male:female ratio was 86:114 (43%/57%); education: elementary school or less / high school / university = 19/115/66 (9.5%/57.5%/33%); employment status: employed / unemployed / pensioner = 57/25/118 (28.5%/12.5%/59%).

All participants had at least one chronic noncommunicable disease; the Charlson Comorbidity Index (CCI) was 3.5 ± 1.8, and the average number of years of disease duration was 9.4 ± 10.6. Systemic medications taken by the subjects included antihypertensives n = 123/200 (61.5%), antidiabetics n = 42/200 (21%), antiplatelet/anticoagulants n = 41/200 (20.5%), antidepressants n = 17/200 (8.5%), thyroid drugs n = 17/200 (8.5%) and others n = 66/200 (33%).

The following habits were recorded in the study sample: active smoker/former smoker/non-smoker 63/45/92 (31.5%/22.5%/46%) with the average number of smoked cigarettes daily amounting to 5.7 ± 9.7, drinking alcohol 73 (36%), drinking coffee 169 (84.5%) with the average number of cups of coffee consumed per day being 2.05 ± 1.4.

The following types of prosthesis were represented in the sample: complete dentures 75 (37.5%), partial acrylic dentures 44 (22%), partial metal prostheses 43 (21.5%), and combination of complete and partial dentures in the maxilla and mandible 38 (19%).

### Reliability Testing

After testing the original 29 items from the questionnaire, and reviewing the results of the correlation matrix, mean values, variance, skewness, and kurtosis of response distributions for each of the items, 1 item was removed, leaving a final version of the OHRQoL-DW scale with 28 items. The removed item had a low correlation with other items (the majority of the Spearman’s correlation coefficients were between 0.098 and 0.280) and with the total score of the remaining 28 items (the correlation coefficient was 0.484, and Cronbach’s alpha remained the same after deletion). Cronbach’s alpha of the final version with 28 items was 0.938 when the scale was rated by the investigators. The mean values of responses, standard deviations, skewness, and kurtosis for each item are shown in Table 1.

**Table 1:** Mean values, standard deviation, skewness and kurtosis of responses to items of OHRQoL-DW

Item	Mean response	Standard deviation	Skewness	Kurtosis
I have difficulties when inserting and removing the prosthesis – Q1	4.1200	1.24634	-1.174	-0.015
I have difficulties maintaining hygiene – Q2	4.3100	1.11360	-1.454	0.754
I’m in pain and discomfort because of dental prostheses – Q3	3.3700	1.41531	-0.141	-1.534
I feel the pain when eating – Q4	3.5350	1.47977	-0.403	-1.436
Food or dentures fall out of my mouth when eating – Q5	4.0800	1.32377	-1.041	-.481
I can’t eat with dentures the food which I have eaten in the past – Q6	3.1200	1.53865	-.036	-1.569
It is hard for me to bite or to chew the food – Q7	2.7950	1.38295	0.454	-1.218
It is hard for me to speak with dentures – Q8	4.0850	1.28297	-1.069	-0.389
I can’t laugh with dentures – Q9	4.5400	0.92340	-20.169	4.168
I can’t kiss a person when I wear dentures – Q10	4.4450	0.93345	-1.486	1.067
The expression of my face does not fit to what I feel – Q11	4.3900	1.05996	-1.498	0.965
I do not like the shape of my artificial teeth – Q12	4.4750	1.04635	-2.086	3.386
I do not like the color of my artificial teeth – Q13	4.5200	1.03681	-2.213	3.860
The denture gives me bad breath – Q14	4.3050	1.10820	-1.321	0.309
I can’t sleep because I have problems with the denture – Q15	4.1550	1.26847	-1.399	0.706
The denture makes me feel uneasy – Q16	3.9400	1.33239	-.829	-0.874
The denture made me lose self-confidence – Q17	4.5750	0.93743	-2.235	4.025
The denture makes me feel depressed – Q18	4.5700	0.95901	-2.290	4.287
The denture makes me feel anxious – Q19	4.2900	1.17165	-1.510	0.972
The denture makes me avoid socializing with friends – Q20	4.8350	0.50899	-3.256	10.273
The denture makes me avoid social events (celebrations, weddings, funerals, birthdays) – Q21	4.6500	0.78138	-2.297	4.672
The denture adversely influenced the relationship with my partner – Q22	4.6150	0.75473	-1.921	3.136
The denture adversely influenced the relationship with my family members – Q23	4.7050	0.73531	-2.536	5.474
The denture makes me avoid leaving my home – Q24	4.7500	0.62406	-2.522	5.539
The denture makes me mind dryness of air in a closed space – Q25	3.6000	1.43187	-0.411	-1.302
The denture makes difficult for me to withstand hot weather – Q26	4.3350	1.00889	-1.276	0.613
The denture makes difficult for me to withstand cold weather – Q27	4.4500	0.91745	-1.370	0.458
The denture makes me avoid swimming (in the sea, lake or swimming pool) – Q28	4.3400	0.97939	-1.084	-0.053

The responses are rated from 1 to 5 on a Likert scale (5: “I disagree completely”; and 1: “I agree completely”); Q: question.

The split-half approach was used to divide the survey into equal halves. The Spearman-Brown “prediction” formula was used to determine the survey’s overall Spearman-Brown coefficient, which had a value of 0.887. The fact that the Spearman-Brown coefficient remained above 0.7 following the split-half approach further supports the questionnaire’s satisfactory reliability (theoretically, this coefficient may take any value between 0 and 1). Cronbach’s alpha was 0.905 when the scale was graded by the patients themselves (during the first encounter).

### Factor Analysis

The principal axis factoring method was used for exploratory factor analysis. The Kaiser-Meyer-Olkin measure of sampling adequacy was 0.890 and the Bartlett’s test of sphericity was statistically significant (p < 0.001). Using generalised least squares extraction after Varimax rotation, four factors were extracted, explaining in total 54.6% of the variance. The first factor bears 4.413 eigenvalues (15.76% of the variance), the second 4.289 (15.32% of the variance), the third 3.310 eigenvalues (11.82% of the variance) and the fourth 3.280 eigenvalues (11.72% of the variance). The rotated factor matrix is shown in Table 2. The items 1, 3-8 and 16 belong to the first factor, which reflects physical aspects of quality of life. The items 9–10, 14–15 and 18-25 belong to factor 2, which describes psychosocial aspects of quality of life, and the items 26-28 describe environmental aspects of quality of life. Aesthetic aspects of quality of life are described by questions 2, 11–13 and 17, which belong to the fourth factor. The four-factor structure is common to other HRQoL instruments, due to the conceptual similarity of four facets of health: physical, psychosocial, aesthetic, and life in relation to the environment.^9^

**Table 2:** The rotated factor matrix of the OHRQoL-DW scale

Item	Factor 1 (Physical aspect of quality of life)	Factor 2 (Psychosocial aspects of quality of life)	Factor 3 (Environmental aspects of quality of life)	Factor 4 (Esthetic aspects of quality of life)
Q1	0.315			
Q2				0.335
Q3	0.747			
Q4	0.815			
Q5	0.485			
Q6	0.776			
Q7	0.597			
Q8	0.408			
Q9		0.504		
Q10		0.459		
Q11				0.445
Q12				0.798
Q13				0.957
Q14		0.488		
Q15		0.550		
Q16	0.529			
Q17				0.536
Q18		0.566		
Q19		0.501		
Q20		0.649		
Q21		0.476		
Q22		0.571		
Q23		0.637		
Q24		0.598		
Q25		0.350		
Q26			0.718	
Q27			0.922	
Q28			0.576	

An item belongs to the factor where its loading is listed. Non-significant loadings are not listed for the sake of clarity. Q: question.

### Validity

Construct validity of the scale was assessed and endorsed by the panel of experts; a few questions were slightly re-phrased by the panel.

Non-parametric correlations between scores of the OHRQoL-DW scale (when it was rated by investigators and by the patients themselves) and scores of the ERQ scale (rated by investigators and patients) were calculated to test divergent criterion validity of the OHRQoL-DW. Non-parametric correlations between scores of the OHRQoL-DW scale (rated by investigators and patients), and scores of the PDT-OHQoL scale (rated by investigators and patients) were used to test convergent criterion validity of the OHRQoL-DW. Non-parametric correlation was chosen due to the non-normal distribution of the majority of the scores. Spearman’s correlation coefficients are shown in the multi-trait, multi-method matrix in Table 3. Spearman’s correlation coefficient is a measure of the strength and direction of association between two variables, based on the rank of individual values instead of actual values; it is a non-parametric correlation coefficient, and its administration does not assume normal distribution of data within the variables.

**Table 3:** Multi-trait, multi-method correlation matrix

	OHRQoL-DW score, rated by investigators	OHRQoL-DW score, rated by patients	PDT-OHQoL score, rated by investigators	PDT-OHQoL score, rated by patients	ERQ score, rated by investigators	ERQ score, rated by patients
OHRQoL-DW score, rated by investigators	1.000					
OHRQoL-DW score, rated by patients	0.901**	1.000				
PDT-OHQoL score, rated by investigators	0.680**	0.636**	1.000			
PDT-OHQoL score, rated by patients	0.642**	0.661**	0.878**	1.000		
ERQ score, rated by investigators	0.089	0.079	-0.028	0.044	1.000	
ERQ score, rated by patients	0.075	0.024	-0.033	-0.014	0.855**	1.000

**p < .01; *p < .05; non-parametric Spearman’s coefficients.

When the OHRQoL-DW scores (taken the 1st, 2nd and 3rd times) were compared between study subjects with different types of dentures (complete, partial acrylic, partial metal and combination of one-jaw complete and partial denture), they were statistically significantly higher in patients with partial metal dentures (Table 4). These results confirm the discriminative ability of the instrument.

**Table 4:** Comparison of OHRQoL-DW total scores (median and interquartile range) across types of dentures

	Complete denture (n = 75)	Partial acrylic denture (n = 44)	Partial metal denture (n = 43)	Combination of one-jaw complete and partial denture (n = 38)	p-value
OHRQoL-DW score, 1st measurement	122 [28]	126.5 [22.5]	134 [15]	129 [29.5]	0.003*
OHRQoL-DW score, 2nd measurement	118 [31]	125.5 [23.3]	132 [13]	126 [30.5]	0.001*
OHRQoL-DW score, 3rd measurement	122 [28]	126.5 [23.3]	135 [15]	128 [30.3]	0.003*

*p < 0.05, Mann-Whitney U-test.

### Temporal Stability

The OHRQoL-DW scale showed excellent temporal stability: when rating by the investigators was repeated on the same patients 15 to 30 days later, the correlation between the scores (Spearman’s coefficient) was 0.957 (p = 0.000). Cronbach’s alpha after the repeated rating was 0.911.

## Discussion

The definitive version of the OHRQoL-DW scale with 28 items showed excellent reliability, with Cronbach’s alpha 0.938. The scale was also temporally stable, and satisfactory results were obtained for divergent and convergent validity tests. Factor analysis discovered four aspects of OHRQoL in patients wearing dentures, physical, psychosocial, environmental and aesthetic. Using this scale, patients wearing different types of removable dentures with a low quality of life related to oral health can be identified, and it can be used for better prosthetic therapy monitoring and improvement of oral health and quality of life.

Currently, among instruments that are adapted to measure quality of life related to oral health in patients wearing dentures, the only one that is primarily used in edentulous patients is OHIP-EDENT, which can detect oral-health-related changes in quality of life in edentulous patients with complete dentures.^1^ The Serbian version of OHIP-EDENT questionnaire tested on 177 complete-denture wearers showed good reliability (Cronbach’s alpha = 0.87).^5^ Another measure of internal consistency of this questionnaire, Guttman’s split-half coefficient, reached a value of 0.74. In our study, the Spearman-Brown coefficient for OHRQoL-DW using the split-half method was 0.87, thus showing very good reliability. OHIP-EDENT results in the Serbian population were similar to those obtained in Brazilian^26^ and Turkish populations,^4^ while Cronbach’s alpha was lower in a Nepalese population.^22^ Results from our questionnaire are similar to those in a Japanese version of OHIP-EDENT (Cronbach’s alpha = 0.93),^21^ Croatian (Cronbach’s alpha = 0.92)^5^ and a Bosnian (Cronbach’s alpha = 0.907)^20^ version, while in a Chinese population it performed better (Cronbach’s alpha = 0.972).^8^ The internal consistency for the different questionnaire domains of original PDT-OHQoL ranged from 0.67 to 0.868.^16^

The temporal stability of our scale was very good; results were obtained 15 to 30 days after first filling in the questionnaire, which is to enough to prevent subjects of remembering the questions. In Serbian and Croatian OHIP-EDENT versions, test-retest reliability was conducted in 30 patients who answered the same questions twice in a 15-day period, with results presented by the intraclass correlation coefficient (ICC), which it was very high in both populations.^5^ In other translated versions of the OHIP instrument, temporal stability was also achieved.^4,21,22^

To identify interrelationships and groupings among items in the OHRQoL-DW, exploratory factor analysis was used. Values obtained from the Kaiser-Meyer-Olkin and Bartlett’s test of sphericity were appropriate to conduct exploratory factor analysis. This analysis extracted four factors or aspects of OHRQoL in denture wearers (physical, psychosocial, aesthetic and life in relation to the environment). The HRQoL recognises six aspects of quality of life: physical, mental, social function, self-integrity, safety, harmony, and spiritual well-being.^28^ Four domains are common in generic instruments that measure HRQoL: physical, psychical, social and environmental.^10^ However, even though the OHRQoL is generally recognised by the scientific literature to be a multidimensional construct that includes physical and psychological factors as well as social well-being, there is no consensus regarding its specific factorial characteristics.^13^ Four domains were extracted from the Serbian and Croatian versions of OHIP-EDENT, with some authors stating that these factors could not be interpreted in a clear manner or could be divided as function, pain, comfort and psychosocial impact.^5^ In the Turkish and Bosnian versions of the same instrument, three domains were extracted: physical impact, psychological and social impact.^4,20^ The Chinese and Nepalese versions of OHIP-EDENT had five extracted factors.^8,22^ The original OHIP-EDENT and the full version of OHIP-49 have seven categories or subscales that were divided into functional limitation, physical pain, psychological discomfort, physical disability, psychological disability, social disability and handicap.^25^ In the pilot study by Mirjitski et al,^16^ in which a constructed-questionnaire PDT-OHQoL explorative factor analysis was not performed, their instrument was divided into six subscales: functional disability, physiological pain, psychological discomfort, physiological disability, psychological disability and social disability.

The validity of the new scale OHRQoL-DW was confirmed by high correlations between summary scores of OHRQoL-DW and accompanying PDT-OHQoL, while there was weak correlation between scores of OHRQoL-DW and ERQ. This is presented in a multi-trait, multi-method correlation matrix. The multi-trait, multi-method analysis was developed by Campbell and Fiske (1959) and is a useful method for assessing construct validity (in particular), convergent validity and discriminant validity.^12^ Other studies did not test the divergent validity of translated questionnaires, while the convergent validity was confirmed by reverse correlation between OHIP-EDENT summary scores and one question in which subjects rated overall satisfaction with their dentures.^4,5^ In the Japanese version of OHIP-EDENT, patients were divided into two groups, a group A in which patients required new dentures and a group B in which patients already had dentures. In this study, content validity was tested by measuring and comparing summary scores between the two groups, while concurrent validity was confirmed by strength of correlation between the summary scores for OHIP-EDENT-J and the degree of satisfaction with dentures (100 mm VAS) for both groups.^21^

The validity of our scale was also tested by comparison of OHRQoL-DW total scores across types of dentures. The patients with removable partial metal dentures had the highest score, i.e., the best quality of life related to oral health. Partial metal dentures are reduced and do not cover all the available tissues of the oral cavity, in contrast to partial acrylic and complete dentures, which are maximally extended. In addition, metal is stronger, which allows these dentures to be thinner and more comfortable for the patient. They also have elements that allow chewing pressure to be transferred to the present teeth, so the load on the mucosa of alveolar ridge is lessened, and the possibility of decubital ulcers is reduced to a minimum.^15^ Furthermore, metal polishes better than acrylic resin, so the accumulation of dental biofilm and microorganisms is lower in partial metal dentures, and maintenance of oral hygiene is better. On the other hand, the loss of all teeth and prosthetic rehabilitation with complete dentures can make it difficult to chew and eat, particularly in elderly populations, despite the attention paid by dentists to promoting oral health. Therefore, it is important to understand concepts and levels of satisfaction in elderly populations regarding oral health and wearing dentures, so dentists may adapt prosthetic interventions to needs of patients.^20^

The main limitation of this study was the use of a convenience sample rather than a random sample to validate the scale, which restricts the ability to draw conclusions that can be generalised. Second, it would be helpful to include individuals who represent the entire spectrum of a phenomenon that is measured and, if possible, validated by a “gold standard” in order to validate a scale. There is still no gold standard for OHRQoL, which leaves room for doubt. Besides, sensitivity and responsiveness of the OHRQoL-DW could not be evaluated, since this would require a longitudinal study.

## Conclusion

The OHRQoL-DW scale is a reliable and valid generic instrument for measuring the oral health-related quality of life in patients wearing various types of conventional dentures; it embraces four aspects of HRQoL: physical, psychosocial, aesthetic and environmental. It could be used not only for research purposes, but also in routine clinical practice for following the quality of life of individual patients, which is one of the most important outcomes of oral health in prosthetic treatment.
